# Cocaine-induced granulomatosis with polyangiitis—an under-recognized condition

**DOI:** 10.1093/rap/rkad027

**Published:** 2023-04-04

**Authors:** Charn Gill, Joseph Sturman, Leyla Ozbek, Scott R Henderson, Aine Burns, Sally Hamour, Ruth J Pepper, Lisha McClelland, Dimitrios Chanouzas, Simon Gane, Alan D Salama, Lorraine Harper

**Affiliations:** Department of ENT Surgery, University Hospitals Birmingham NHS Foundation Trust, Birmingham, UK; Renal Unit, University Hospitals Birmingham NHS Foundation Trust, Birmingham, UK; Department of ENT surgery, Royal National ENT Hospital, University College London Hospital, London, UK; UCL Department of Renal Medicine, , Royal Free Hospital, London, UK; Renal Unit, University Hospitals Birmingham NHS Foundation Trust, Birmingham, UK; Renal Unit, University Hospitals Birmingham NHS Foundation Trust, Birmingham, UK; UCL Department of Renal Medicine, , Royal Free Hospital, London, UK; Department of ENT Surgery, University Hospitals Birmingham NHS Foundation Trust, Birmingham, UK; Department of ENT Surgery, University Hospitals Birmingham NHS Foundation Trust, Birmingham, UK; Institute Applied Health Research, College of Medical and Dental Sciences, University of Birmingham, Birmingham, UK; Department of ENT surgery, Royal National ENT Hospital, University College London Hospital, London, UK; UCL Department of Renal Medicine, , Royal Free Hospital, London, UK; Department of ENT Surgery, University Hospitals Birmingham NHS Foundation Trust, Birmingham, UK; Institute Applied Health Research, College of Medical and Dental Sciences, University of Birmingham, Birmingham, UK

**Keywords:** cocaine, drug-induced vasculitis, granulomatosis with polyangiitis, ANCA, treatment

## Abstract

**Objectives:**

Cocaine and cocaine mixed with levamisole are increasingly used in the UK and result in significant direct nasal damage in addition to promoting vasculitis. Our aims were as follows: (1) to identify the main symptoms and presentation of cocaine-induced vasculitis; (2) to provide evidence regarding the best practice for the investigation and diagnosis of cocaine-induced vasculitis; and (3) to analyse the clinical outcomes of patients in order to understand the optimal management for the condition.

**Methods:**

We performed a retrospective case series analysis of patients presenting with cocaine-induced midline destructive lesions or vasculitis compatible with granulomatosis with polyangiitis (GPA) from two large tertiary vasculitis clinics between 2016 and 2021.

**Results:**

Forty-two patients (29 Birmingham, 13 London) with cocaine-induced midline lesions or systemic disease were identified. The median age was 41 years (range 23–66 years). Current cocaine use was common, and 20 of 23 samples provided were positive when routine urine toxicology was performed; 9 patients who denied ever using cocaine were identified as using cocaine based on urine toxicology analysis, and 11 who stated they were ex-users still tested positive. There was a high incidence of septal perforation (75%) and oronasal fistula (15%). Systemic manifestations were less common (27%), and only one patient had acute kidney injury. Fifty-six per cent of our patients were PR3-ANCA positive, with none testing positive for MPO-ANCA. Symptom remission required cocaine discontinuation even when immunosuppression was administered.

**Conclusion:**

Patients with destructive nasal lesions, especially young patients, should have urine toxicology performed for cocaine before diagnosing GPA and considering immunosuppressive therapy. The ANCA pattern is not specific for cocaine-induced midline destructive lesions. Treatment should be focused on cocaine cessation and conservative management in the first instance in the absence of organ-threatening disease.

Key messages

- Cocaine toxicology should be performed especially in young patients with a diagnosis of limited granulomatosis with polyangiitis.
- ANCA is common, but the subtype is not specific for cocaine-induced nasal disease.
- Treatment should focus on cocaine abstinence rather than immunosuppression.

## Introduction

Cocaine is the second most commonly abused drug in the UK [1], with 2.6% of the population aged between 16 and 59 years old using it. It is known to cause significant pathology, including cocaine-induced midline destructive lesions [2] and various vasculitic lesions [3]. Over the last 30 years, there is a growing body of evidence showing that cocaine triggers the production of ANCA, leading to a clinical presentation which can mimic idiopathic granulomatosis with polyangiitis (GPA) [2, 4–6]. It remains unclear whether cocaine itself is the main instigator of the observed pathological process or whether the anti-helminthic drug levamisole, a compound commonly mixed with cocaine, is the true culprit [3]. In the USA, 60% of cocaine is adulterated with levamisole, and this figure is believed to be as high as 80% in Germany [7]. However, both cocaine and levamisole have been shown independently to cause a vasculitis, and both might be implicated in disease pathogenesis [3, 8–10].

The literature consists of case reports and case series with small populations of patients. The most common presentations of cocaine/levamisole-induced vasculitis are with midline destructive lesions and cutaneous vasculitis, often presenting as a retiform purpuric rash predominantly affecting the face and lower limbs [3, 11, 12]. Pulmonary and renal involvement has been reported but is rarer than the cutaneous and nasal manifestations [7, 13–15]. Although the data are limited, palatal perforation is reported as being seen only in patients who abuse cocaine and not observed in those with idiopathic GPA [11].

The reported ANCA patterns seen with cocaine-induced disease are variable, although dual PR3- and MPO-ANCA positivity has been suggested to be pathognomonic of cocaine-induced disease [16]. Anti-human neutrophil elastase antibodies have also been postulated to be strongly associated with cocaine-induced vasculitis, but routine testing for anti-human neutrophil elastase antibodies is not widely available in the UK [9].

Consequently, there remains ongoing debate about the optimal evidence-based diagnosis and management of this patient population. In this retrospective study, we report outcomes from our cohort of 42 patients to contribute additional data in order to enhance our understanding of this condition.

## Methods

A retrospective review was carried out of patients presenting between 2016 and 2021 to the joint vasculitis clinic at Queen Elizabeth Hospital, Birmingham and at the Royal Free Hospital, London who disclosed cocaine use or had positive urine toxicology for cocaine with symptoms suggestive of ANCA-associated vasculitis. Data were collected retrospectively through electronic medical records. The data collected included clinical manifestations, ANCA serology by both ELISA and IIF, urine toxicology, nasal tissue histology and further management. Urine toxicology is routinely requested from patients at the Birmingham clinic on all patients referred. Urine toxicology is performed on an *ad hoc* basis dependent on levels of suspicion of cocaine use in the London clinic.

Analysis was done as part of service evaluation, registered within UHB NHS Foundation Trust under CARMS-17880. Application of the NHS HRA research decision aid toolkit confirmed that research ethics committee approval was not required.

## Results

A total of 42 patients were identified: 23 males, 18 females and 1 patient who did not identify with either gender; 13 patients from the London and 29 from the Birmingham clinics. The median age was 41 years (range 23–66 years old). The baseline demographics are shown in Table 1.

**Table 1.** Characteristics of the patient population

Characteristic	Birmingham (*n* = 29)	London (*n* = 13)
Age, median (range), years	38 (23–57)	49 (32–66)
Sex (M:F:N)	16:12:1	7:6:0
Ethnicity		
Asian	3	0
Caucasian	26	9
Black	0	0
Unknown	0	4
ANCA		
PR3-positive ELISA	17	7
MPO-positive ELISA	0	0
IIF only	8	1
pANCA by IIF and PR3	5	2
cANCA by IIF and PR3	8	1
Negative by IIF or ELISA	2	3
Not checked	2	0
Creatinine, median (range), µmol/l	74 (50–120)	77 (59–220)

F: female; M: male; N: not declared.

All patients were asked specifically about illicit drug use; 11 patients confirmed current cocaine use, 22 admitted to cocaine usage in the past, and 9 patients denied all cocaine use (all Birmingham). Urine toxicology is requested routinely from patients at the Birmingham clinic; 25 patients had urine toxicology performed, of whom 22 (88%) tested positive for cocaine, 9 patients denied ever using and 11 reported as ex-users. Of those who tested positive, 12 individuals also tested positive for other illicit drugs or drugs of abuse, including five cannabis, four codeine, three benzodiazepines, two morphine and one levamisole. Five patients had two or more illicit substances detected in their urine in addition to cocaine. The three patients who tested negative for cocaine all confirmed that they were previous users. Of the four patients who did not provide a urine sample for toxicology, three patients confirmed previous usage but denied current use, and one stated they were currently using cocaine. Urine toxicology was performed on an *ad hoc* basis in the London cohort and was undertaken in nine patients, of whom seven had a positive test and confirmed current usage, while two patients confirmed previous usage and had negative urine toxicology. Of the patients from London who tested positive for cocaine, six patients also tested positive for other drugs, including three positive for opiates and four positive for cannabis.

All bar one patient had nasal symptoms. On clinical examination of those with nasal symptoms, 30 patients had evidence of septal perforation. On further examination, six patients had oronasal fistulas. Twelve patients (27%) had other systemic manifestations, which included skin lesions or rashes, joint pain, breathlessness, fatigue, diplopia and night sweats. None of our patients had pulmonary haemorrhage, and median creatinine was 76 µmol/l (range 50–220 µmol/l). Two patients had a renal biopsy owing to haematuria, with only one patient having evidence of inflammation consistent with IgA vasculitis. This patient was the only patient with acute kidney injury (creatinine 220 µ mol/l) and had no ENT symptoms. He denied current cocaine use, with no urine toxicology performed to confirm this.

Forty patients (92.9%) had blood tests for ANCA to include MPO and PR3 antibodies measured by ELISA and cANCA and pANCA patterns measured by IIF; 87.5% (*n* = 35) of individuals were positive by ELISA and/or IIF. Twenty-four patients (56%) tested positive for PR3 antibodies and none was positive for MPO by ELISA. Twenty-eight patients had positive ANCA results by IIF; of these, 1 was an atypical result, 11 patients were positive for cANCA and 12 for pANCA, there were 3 patients who were positive for both cANCA and pANCA and 1 reported as positive without a pattern. PR3-ANCA is usually associated with an IIF cANCA pattern; however, only nine patients positive for PR3 via ELISA had an associated cANCA pattern by IIF (37.5%) and seven (29%) PR3-positive patients were positive for p-ANCA. Four PR3-positive patients reported both cANCA and pANCA. Six PR3-positive patients were negative by IIF or borderline positive.

Twenty-eight patients had a nasal or sinus biopsy, none of whom showed evidence of granulomatous inflammation, although two patients had evidence of small vessel vasculitis.

Treatment between the two centres differed. All patients were informed of the risks of ongoing cocaine use and advised to stop. At Birmingham, patients were specifically advised that immunosuppressive treatment in the absence of organ-threatening disease or nasal reconstruction was dependent on negative urine toxicology on at least two occasions. All patients were advised to commence nasal douching. Twelve of 28 patients (42.9%) were prescribed Co-trimoxazole antibiotics, and 13 of 28 (46.4%) patients were advised to use emollients. In London, patients were advised to stop using cocaine, but there was no requirement for negative toxicology before using immunosuppression and this was given according to physician preference.

Eight patients referred to the Birmingham centre had previously been given a diagnosis of GPA, with a further two patients having a differential of GPA at other centres. Of the eight patients previously diagnosed with GPA, all had been treated with immunosuppression with either one or more of rituximab, azathioprine (AZA), methotrexate (MTX), cyclophosphamide (CYC) or mycopheonlate mofetil (MMF); despite this treatment they had ongoing active disease.

With this management approach, patient follow-up of Birmingham patients was challenging. Twenty-two patients (50%) were lost to follow-up (19 did not attend and 3 were discharged back to the referring site). Seven of 29 (21%) patients remained under the care of the Birmingham joint vasculitis clinic, three of whom continue to have positive urine toxicology and symptoms, three patients have stopped cocaine usage and symptoms have settled, and one patient reports stopping but has persistent symptoms; no urine toxicology has been performed to confirm abstinence.

Of the 13 patients treated in London, two were treated conservatively, one of whom stopped using cocaine and symptoms settled. Three patients were treated with oral prednisolone. All three continued to have symptoms, although there was some partial improvement. Two of these three patients reported persistent use of cocaine. Seven patients received prednisolone and rituximab, of whom four improved, including the patient with renal disease. All four patients who improved reported stopping cocaine usage. Three of the seven patients who were treated with prednisolone and rituximab reported no improvement, but all three continued to use cocaine. Finally, one patient received MTX for RA but was lost to follow-up.

## Discussion

This study represents the largest cohort of patients with cocaine-induced vasculitis in the UK, and our findings make a significant contribution to the existing literature. Using a systematic approach in our Birmingham clinic, we identified 28 patients over a 5-year period who presented with symptoms compatible with GPA associated with cocaine usage. Nine of these patients denied any cocaine use. Failure to undertake urine toxicology would have resulted in a missed diagnosis in 32% of our patients, who probably would have gone on to receive treatment with immunosuppression. Using a high index of suspicion, our London colleagues diagnosed 13 patients over the same length of time. Both these approaches reveal considerably higher rates of diagnosis than previously published [17]. Patients with cocaine-induced mimics of GPA are considerably younger, with a median age of 41 years; this is 20 years younger than patients identified with idiopathic GPA and predominantly ENT symptoms [18].

ANCA positivity is well recognized in patients with cocaine-induced disease; only five of our patients were negative for ANCA by either IIF or ELISA. However, as is well described, ANCA positivity is not diagnostic of ANCA-associated vasculitis. Indeed only 2 of 16 patients who had a nasal or sinus biopsy had evidence of vasculitis, and none had granulomata characteristic of GPA.

Our patients were characteristic of patients with cocaine-induced midline destructive lesions, with 30 patients having septal perforations, of whom six had oronasal fistulas. Patients with midline destructive lesions are reported to be PR3-ANCA positive, with a typical pANCA pattern [9]. However, we found that only 29% of our patients had a pANCA pattern with PR3-ANCA positive by ELISA. In a study of 30 patients from the USA, where 70% of cocaine is adulterated with levamisole, MPO-ANCA were reported in all cases, and 50% were dual positive for both MPO and PR3; they considered dual positivity to be pathognomonic of cocaine-induced disease [19]. An additional prospective cohort study comparing 31 patients with cocaine/levamisole-induced vasculitis and 45 patients with idiopathic vasculitis found that the cohort using cocaine were more likely to be MPO positive (100% *vs* 67%) [20]. However, none of our patients had MPO-ANCA identified by ELISA. Interestingly, 6 of 149 (4%) patients who were IIF positive from the CYCLOPS trial tested positive for both PR3- and MPO-ANCA [21]. All these patients had renal involvement, which is uncommon in cocaine-induced disease, adding weight to our results that dual positive ANCA is not pathognomonic of cocaine-induced pseudo-vasculitis.

Although our findings are similar to those of a previous publication from London [17], results might differ from other publications because the amount of levamisole present in samples from our patients is unknown. A European analysis suggested that 40–70% of cocaine is contaminated with levamisole but at variable concentrations, with the average cocaine sample analysed containing 50% cocaine and 8% levamisole [22]. This might impact on the autoantibodies detected. Cocaine-induced vasculitis can present with constitutional and systemic symptoms. However, only 28% of our patients had symptoms beyond the upper respiratory tract. Only one of our patients had acute kidney injury, but there was no pulmonary haemorrhage. Our results might differ from other published studies because the majority of our patients were diagnosed through a specialized ENT/vasculitis service and presented with cocaine-induced midline destructive lesion-predominant disease, reflecting the referral pathways, whereas other publications are predominantly from a rheumatological or nephrological pathway [17, 19]. Palatal perforation is rare in idiopathic GPA [11], but 15% of our patients had oronasal fistula. Our data support the view that patients with destructive nasal lesions without significant systemic symptoms should be investigated for cocaine-induced disease, especially if younger than expected.

### Treatment approaches

There is significant heterogeneity in the literature regarding treatment of patients with cocaine-induced vasculitis. Corticosteroids (CSs) and immunosuppression with MTX and CYC are often used [17]. The practice in our Birmingham centre is to offer immunosuppressive therapy if the patient has stopped using cocaine and remains symptomatic. Importantly, patients who continued to be followed up and managed to discontinue cocaine use improved without the need for immunosuppression. Our London colleagues have used immunosuppression in the majority of patients. Despite this, patients failed to eradicate symptoms without stopping cocaine use. The experience in our two different centres suggests that discontinuation of cocaine is required to manage patients and that symptoms will persist despite immunosuppression if there is ongoing cocaine use. This is in agreement with case reports in the literature [3, 5, 12, 23–25, 26]. Numerous studies have observed that cessation of cocaine use leads to complete resolution of the condition [3, 23–25]. Furthermore, where immunosuppressive therapies, such as CSs or CYC, were used (mainly in cases of renal involvement), full resolution was not achieved unless the patient stopped using cocaine [25, 27]. In patients who did not stop using cocaine, resolution was never achieved [13, 14]. Immunosuppression is associated with significant risks, particularly infection, and inappropriate use can cause significant harm. Given our evidence of lack of benefit, immunosuppression in the absence of failure to discontinue cocaine should be considered very carefully, and we would suggest not used. Individuals should be referred to addiction counselling services to help with cocaine cessation.

There is evidence to suggest that autoantibody levels normalize within 2–14 months of discontinuing cocaine/levamisole without immunosuppressive treatment [23]. This might reflect laboratory research showing that levamisole activates neutrophils via M_3_ muscarinic acetylcholine receptors to extrude their intracellular proteins in neutrophil extracellular traps (NETs) via a process called NETosis, triggering autoantibody formation [10]. Withdrawal of levamisole led to cessation of NETosis and a reduction in autoantibody production without the need for other interventions [10].

### Strengths and limitations

This report describes the largest cohort of patients in the UK with cocaine-induced vasculitis and emphasizes the importance of performing toxicology on patients who present with isolated nasal symptoms that might be consistent with vasculitis. The different treatment approaches between the two centres allows us to compare outcomes between the two strategies, emphasizing the need for cocaine discontinuation and conservative management.

There were limitations to the study, including its retrospective nature and a high proportion of patients lost to follow-up. We used urine toxicology testing routinely in only one centre, which might therefore underestimate the number of patients with this diagnosis. Given that the majority of patients came via an ENT referral pathway, this might skew the number of patients with the cocaine-induced midline destructive lesions pattern of disease, with under-representation of those with more systemic disease. We were unable to report the longitudinal evaluation of how ANCA levels or binding patterns changed owing to the high proportion of patients lost to follow-up.

### Conclusion

Our data show that cocaine-induced vasculitis is more common than first reported and that toxicology should be considered on all patients who appear to have isolated nasal involvement with vasculitis. We argue that MPO-ANCA positivity might not be as common as previously reported and that dual positive ANCA is uncommon, and therefore, the lack of dual positive PR3- and MPO-ANCA should not be used to exclude cocaine-induced disease. We are reassured to report that renal and pulmonary involvement appears to be rare. Furthermore, we advocate for cessation of cocaine use as the first strategy, without use of immunosuppression, for the management of these patients.

## Data Availability

The data underlying this article are available in the article.
